# Is the Negro Subject to Hare-Lip?

**Published:** 1845-09

**Authors:** John Harris

**Affiliations:** Annapolis


### ARTICLE VI.

### *Is the Negro subject to Hare-lip?*

MESSRS. EDITORS:

In the last number of the "American Journal and Library of Dental Science," 5th volume, page 314, is an extract from the "Western Lancet," headed by the interrogatory—"Is the negro subject to hare-lip?"

Of the thousands of negroes the author had seen in the slave and free states, not one, of any complexion, had hare-lip, and that the observation of gentlemen, who had resided years in slave states, with whom he had conversed, coincided with his own. He adds, "that in his own district, and elsewhere, the deformity forces itself upon him very frequently, but is exclusively confined to the whites," and asks the question—"Is this disparity in the races generally throughout the United States, and, if so, is there a philosophical reason for the difference?'

The inquiry is put to the profession, and their attention is respectfully solicited to it.

A circumstance occurred this morning, which very forcibly reminded me of having read the article while at my brother's house in Baltimore, and of his asking me at the time if I had ever seen a negro who had hare-lip; to which, upon reflecting, I responded in the negative; and but for the circumstance referred to, I know not how long the impression left upon my mind might have lasted.

On arriving at this place, I called at the residence of Dr. Ridout, and, not finding him at home, left my address.

This morning I was favored with a call at my room from the Doctor, and while with me, a part of his time was occupied in examining the Dental Journal. I soon perceived that he had discovered something that appeared to amuse him very much, and found it to be the extract alluded to. He then referred me to a negro woman in this city, and a negro man in the immediate vicinity, who had hare-lip. The woman I have seen. The Doctor also informed me that he had seen several other negroes laboring under the same deformity.

If the conclusions arrived at by the author were true, it would not be more strange nor difficult to account for, than that females are almost exclusively the subjects of bronchocele, or goitre, (commonly called the big neck in females,) than nevea materni, or other phenomena connected with the family of luses naturæ, are occasionally to be met with.

Since my interview with Dr. R., I have heard of several other negroes who have hare-lip.

Respectfully, &c.

JOHN HARRIS.

*Annapolis, July 14th*, 1845.

